# Supplementary material for: Coding Sequence Insertions in Fungal Genomes are Intrinsically Disordered and can Impart Functionally-Important Properties on the Host Protein
Source: bioRxiv. 2023 Apr 7:2023.04.06.535715. Preprint. [Version 1] doi: 10.1101/2023.04.06.535715 (PMC10104129; doi:10.1101/2023.04.06.535715)
Supplement: Supplement 1 [file media-1.pdf]

CTG strains	No. CDIs	Saccharomycetaceae strains	No. CDIs	Aspergillaceae strains	No. CDIs	Herpotrichiellaceae strains	No. CDIs
Candida albicans	1366	Candida glabrata	1466	Aspergillus aculeatus	226	Capronia coronata	431
Candida auris	1332	Eremothecium cymbalariae	1319	Aspergillus clavatus	221	Capronia epimyces	439
Candida dubliniensis	1364	Eremothecium gossypii	1325	Aspergillus fischeri	220	Cladophialophora bantiana	495
Candida duobushaemulonis	1295	Kazachstania africana	1472	Aspergillus flavus	235	Cladophialophora carrionii	493
Candida haemuloni	1327	Kazachstania naganishii	1468	Aspergillus fumigatus	219	Cladophialophora immunda	480
Candida orthopsilosis	1310	Kluyveromyces lactis	1362	Aspergillus nidulans	237	Cladophialophora psammophila	494
Candida pseudohaemulonii	1316	Kluyveromyces marxianus	1358	Aspergillus niger	207	Cladophialophora yegresii	483
Candida tropicalis	1296	Lachancea lanzarotensis	1349	Aspergillus nomiae	232	Exophiala aquamarina	427
Clavispora lusitaniae	1266	Lachancea thermotolerans	1350	Aspergillus oryzae	223	Exophiala dermatitidis	424
Debaryomyces fabryi	1273	Naumovozyma castellii	1498	Aspergillus ruber	218	Exophiala mesophila	413
Debaryomyces hansenii	1309	Naumovozyma dairenensis	1512	Aspergillus sydowii	247	Exophiala oligosperma	427
Hyphopichia burtonii	1336	Saccharomyces cerevisiae	1514	Aspergillus terreus	225	Exophiala spinifera	433
Lodderomyces elongisporus	1261	Saccharomyces eubayanus	1506	Penicilliopsis zonata	224	Exophiala xenobiotica	425
Metschnikowia bicuspidata	1299	Saccharomyces paradoxus	1503	Penicillium arizonense	264	Fonsecaea erecta	490
Meyerozyma guilliermondii	1225	Tetrapisispora blattae	1449	Penicillium digitatum	290	Fonsecaea monophora	489
Scheffersomyces spartinae	1228	Tetrapisispora phaffii	1482	Penicillium expansum	289	Fonsecaea multimorphosa	488
Scheffersomyces stipitis	1359	Torulaspora delbrueckii	1453	Penicillium griseofulvum	289	Fonsecaea nubica	490
Spathaspora passalidarum	1274	Vanderwaltozyma polyspora	1421	Penicillium roqueforti	289	Fonsecaea pedrosoi	490
Suhomyces tanzawaensis	1336	Zygosaccharomyces rouxii	1441	Penicillium rubens	289	Phialophora attinorum	347
Yamadazyma tenuis	1221	Zygotorulaspora mrakii	1468	Penicillium solitum	293	Rhinocladiella mackenziei	442
CDIs shared by all strains	640<br>(39.3%)	CDIs shared by all strains	881<br>(50.5%)	CDIs shared by all strains	199<br>(55.3%)	CDIs shared by all strains	291<br>(52.4%)

Supplementary Table 1. Number of CDIs retained by each strain of the four taxonomic families. CDIs were counted as retained if at least one amino acid was present in the length of the insertion event. The highest and lowest numbers of retained CDIs are highlighted for each clade in green and red, respectively. The number of CDIs retained by all 20 strains of each clade is also indicated. The number of CDIs retained as a fraction of the total number of CDIs in the clade is presented as a percentage.

Strain	*C. albicans*	*S. cerevisiae*	*A. oryzae*	*C. coronata*
inserts total	3,069	3,193	1,261	1,782
insert length 1-5	2,056	2,154	835	1,182
insert length 6-10	449	463	223	306
insert length 11-20	314	314	143	198
insert length 21-50	202	219	54	83
insert length >50	48	43	6	13
insert length >100	8	9	1	4
no. of MSAs	1,015	1,064	623	736
inserts per MSA	3.0	3.0	2.0	2.4
% MSAs with inserts	66.3	69.5	40.7	48.0

Supplementary Table 2. Number of inserts present in one strain from each clade. Inserts in each of the four strains were identified and grouped by length. The total number of MSAs analyzed is 1,532 and the number of MSAs in which all inserts are found is indicated. Only MSAs with inserts are included in the calculation of inserts per MSA.

pLDDT score	No. of residues	Percent
10-20	17	0.1
20-30	3,164	14.5
30-40	6,918	31.7
40-50	2,734	12.5
50-60	1,823	8.4
60-70	1,466	6.7
70-80	1,541	7.1
80-90	2,196	10.1
90-100	1,952	8.9
**Total**	**21,811**	**100.0**

Supplementary Table 3. Number of insert residues in each pLDDT score range. The pLDDT scores for 21,811 residues from 3,013 inserts were extracted and number of residues in each score range tabulated.

Insert length	No. of inserts	Average pLDDT score	No. of residues
1-10	2,458	66.04	7,591
11-20	308	48.17	4,477
21-30	112	44.80	2,778
31-40	60	39.95	2,085
41-50	27	39.76	1,223
51-100	40	40.25	2,591
101-150	8	47.40	1,066
**Total**	**3,013**	**51.72**	**21,811**

Supplementary Table 4. Average pLDDT scores by insert length. The pLDDT scores for 21,811 residues from 3,013 inserts were extracted and average pLDDT score for each insert range tabulated.

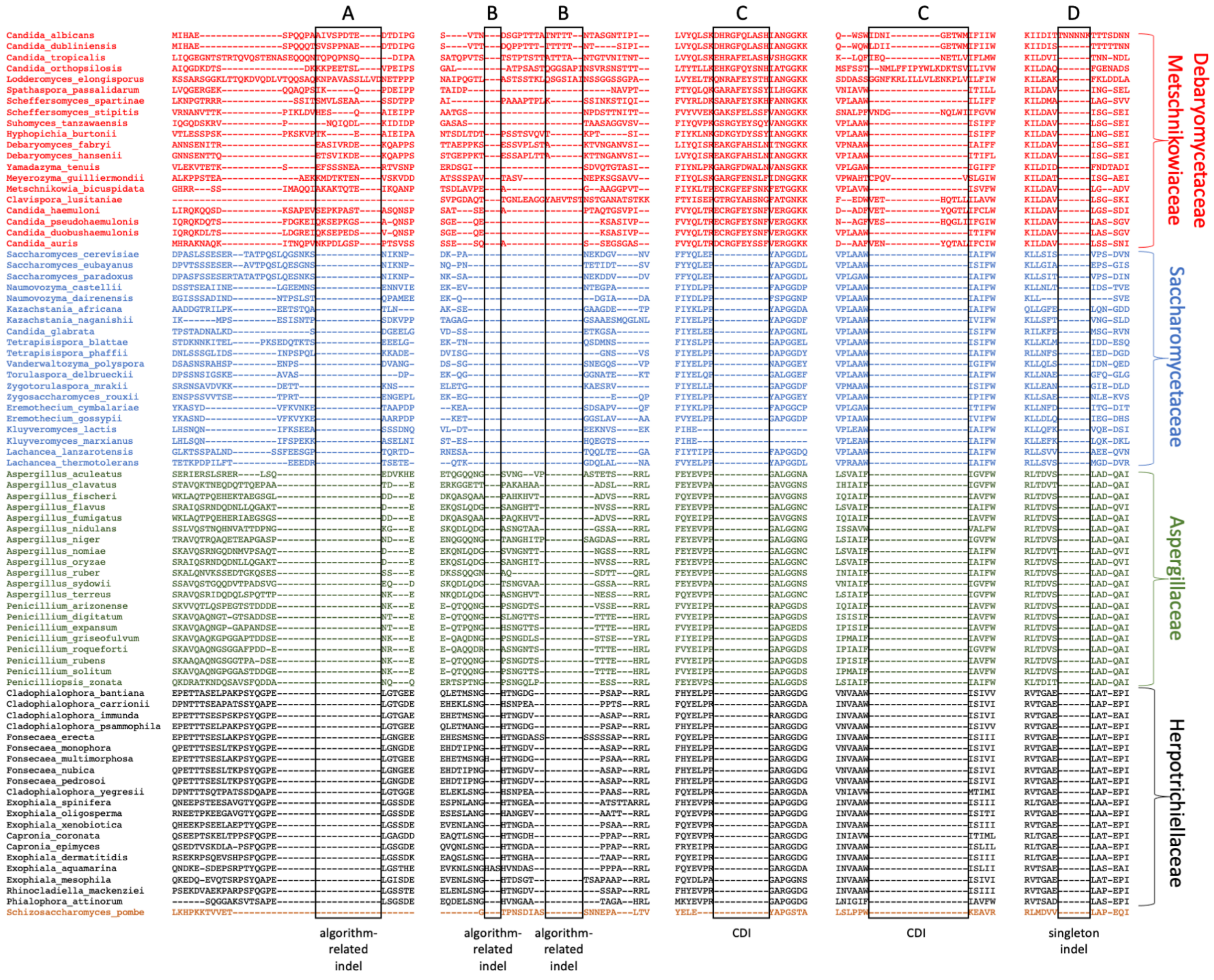

A
B
B
C
C
D
Candida_albicans
Candida_dubliniensis
Candida_tropicalis
Candida_orthopsilosis
Lodderomyces_elongisporus
Spathaspora_passalidarum
Scheffersomyces_spartinae
Scheffersomyces_stipitis
Suhomyces_tanzawaensis
Hyphopichia_burtonii
Debaryomyces_fabryi
Debaryomyces_hansenii
Yamadazyma_tenuis
Meyerozyma_guilliermondii
Metschnikowia_bicuspidata
Clavispora_lusitaniae
Candida_haemuloni
Candida_pseudohaemulonis
Candida_duobushaemulonis
Candida_auris
Saccharomyces_cerevisiae
Saccharomyces_eubayanus
Saccharomyces_paradoxus
Naumovozyma_castellii
Naumovozyma_dairenensis
Kazachstania_africana
Kazachstania_naganishii
Candida_glabrata
Tetrapisispora_blattae
Tetrapisispora_phaffii
Vanderwaltozyma_polyspora
Torulaspora_delbrueckii
Zygotorulaspora_mrakii
Zygosaccharomyces_rouxii
Eremothecium_cymbalariae
Eremothecium_gossypii
Kluyveromyces_lactis
Kluyveromyces_marxianus
Lachancea_lanzarotensis
Lachancea_thermotolerans
Aspergillus_aculeatus
Aspergillus_clavatus
Aspergillus_fischeri
Aspergillus_flavus
Aspergillus_fumigatus
Aspergillus_nidulans
Aspergillus_niger
Aspergillus_nomiae
Aspergillus_oryzae
Aspergillus_ruber
Aspergillus_sydowii
Aspergillus_terreus
Penicillium_arizonense
Penicillium_digitatum
Penicillium_expansum
Penicillium_griseofulvum
Penicillium_roqueforti
Penicillium_rubens
Penicillium_solitum
Penicilliopsis_zonata
Cladophialophora_bantiana
Cladophialophora_carrionii
Cladophialophora_immunda
Cladophialophora_psammophila
Fonsecaea_erecta
Fonsecaea_monophora
Fonsecaea_multimorphosa
Fonsecaea_nubica
Fonsecaea_pedrosoi
Cladophialophora_yegresii
Exophiala_spinifera
Exophiala_oligosperma
Exophiala_xenobiotica
Capronia_coronata
Capronia_epimyces
Exophiala_dermatitidis
Exophiala_aquamarina
Exophiala_mesophila
Rhinocladiella_mackenziei
Phialophora_attinorum
Schizosaccharomyces_pombe
Debaryomycetaceae
Metschnikowiaceae
Saccharomycetaceae
Aspergillaceae
Herpotrichiellaceae
algorithm-related indel
algorithm-related indel
algorithm-related indel
CDI
CDI
singleton indel

Supplementary Figure 1A. Examples of inserts. Five partial alignments are shown. (**A,B**) Algorithm-related inserts are at least partly generated from matrix scoring and/or gap insertion parameters of the algorithm. (**C**) Clade-defining inserts (CDIs) have inserts restricted to a single clade. The CDI must have at least one position with 5 or more aligned amino acids, but otherwise may contain gaps. CDIs must have well-aligned flanking positions with 59 or more residues on both sides of the indel. (**D**) Singleton inserts are found in only one sequence.

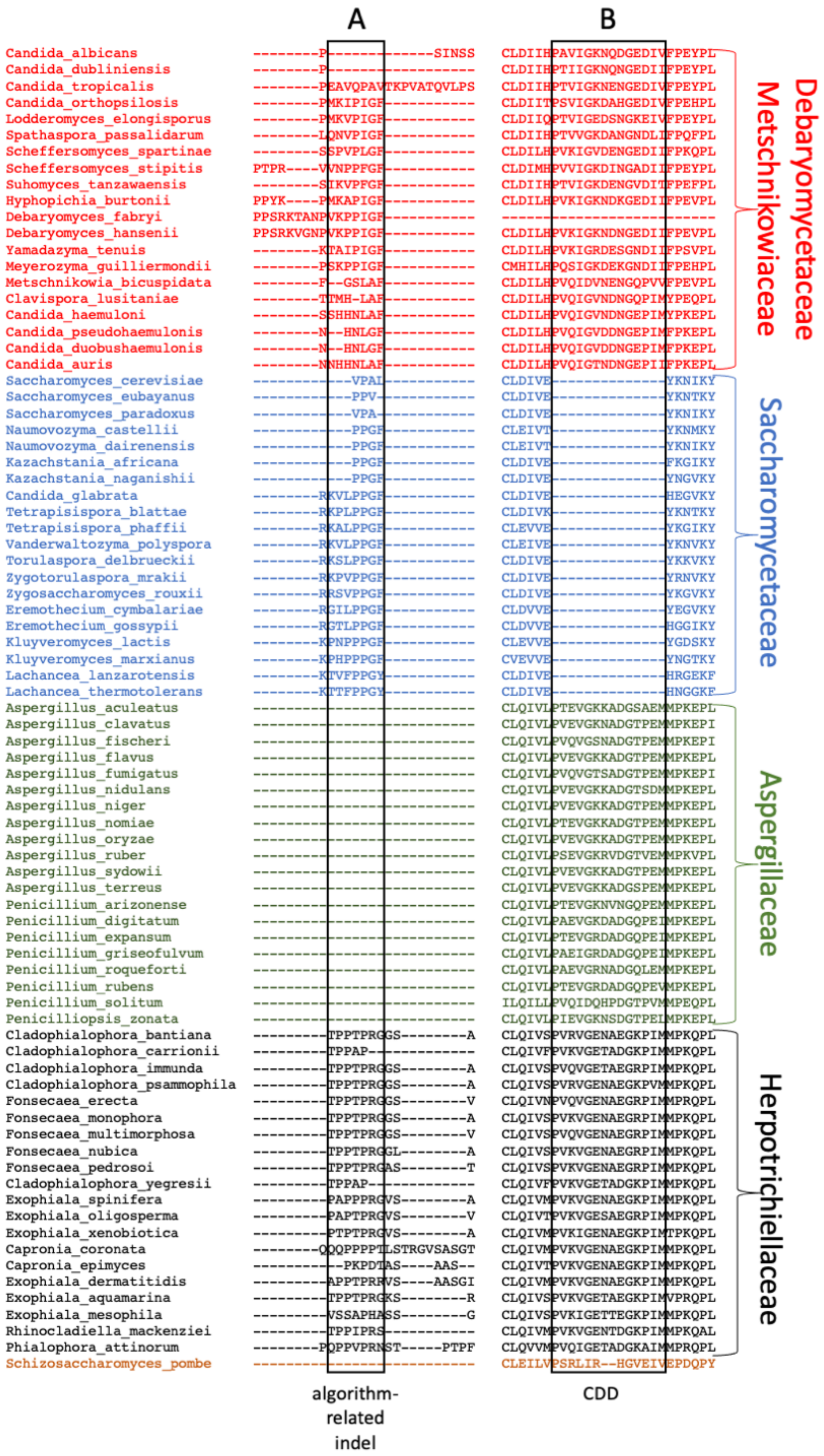

A
B
Debaryomycetaceae
Metschnikowiaceae
Saccharomycetaceae
Aspergillaceae
Herpotrichiellaceae
algorithm-related indel
CDD

Supplementary Figure 1B. Examples of deletions. Two partial alignments are shown. (**A**) An algorithm-related deletion in Aspergillaceae likely arose from the insertion of gaps into a poorly aligning region of the MSA. (**B**) Clade-defining deletions (CDDs) have deletions restricted to a single clade, here in Saccharomycetaceae. The CDD must have at least 5 amino acids in each of the three other clades, but otherwise may contain gaps. CDDs must have flanking positions with 40 or more residues on both sides.

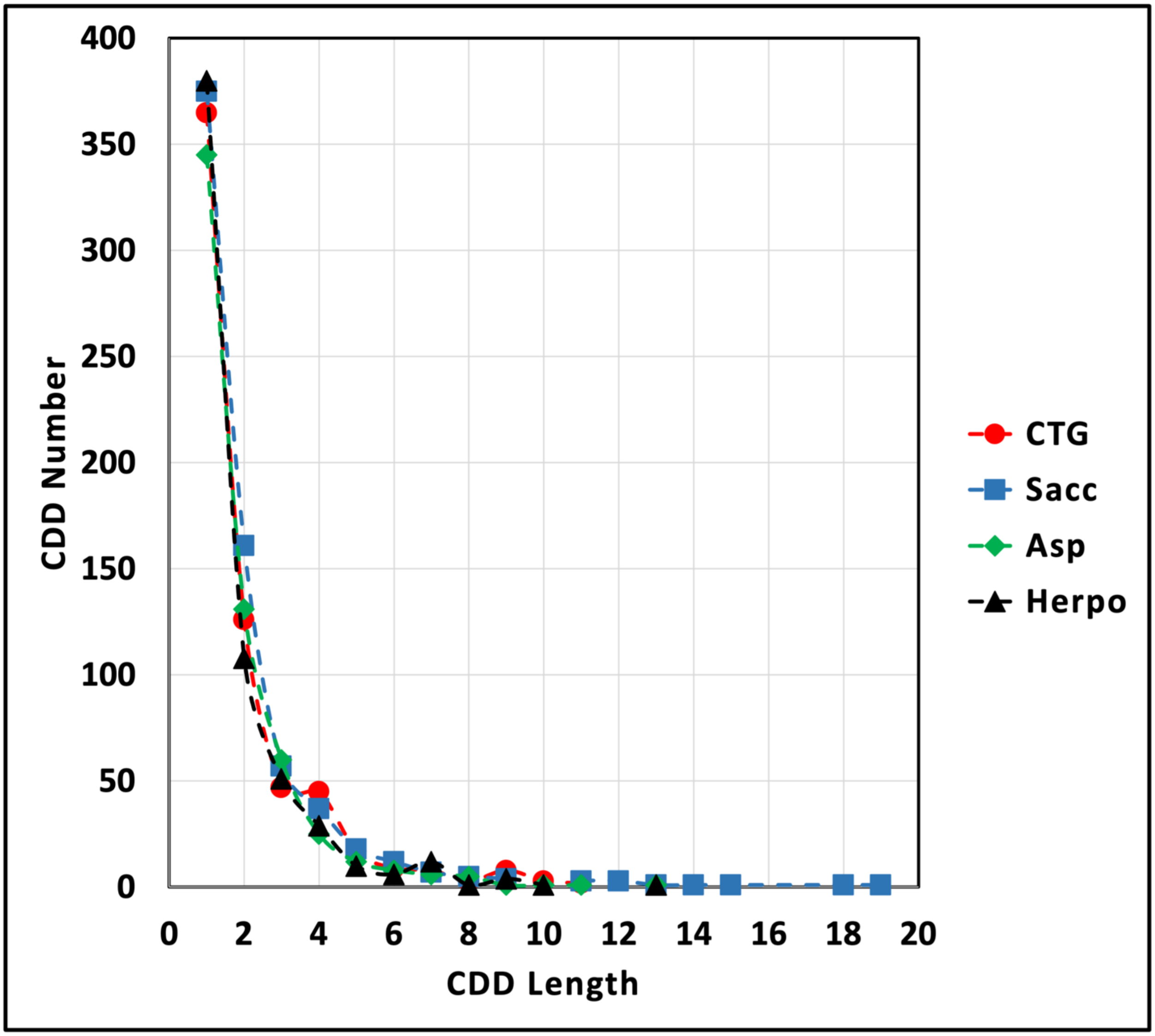

Supplementary Figure 2A. Length distribution of CDDs. The numbers of CDDs were plotted for each clade. Red circles, CTG clade; blue squares, Saccharomycetaceae clade; green diamonds, Aspergillaceae clade; black triangles, Herpotrichiellaceae clade.

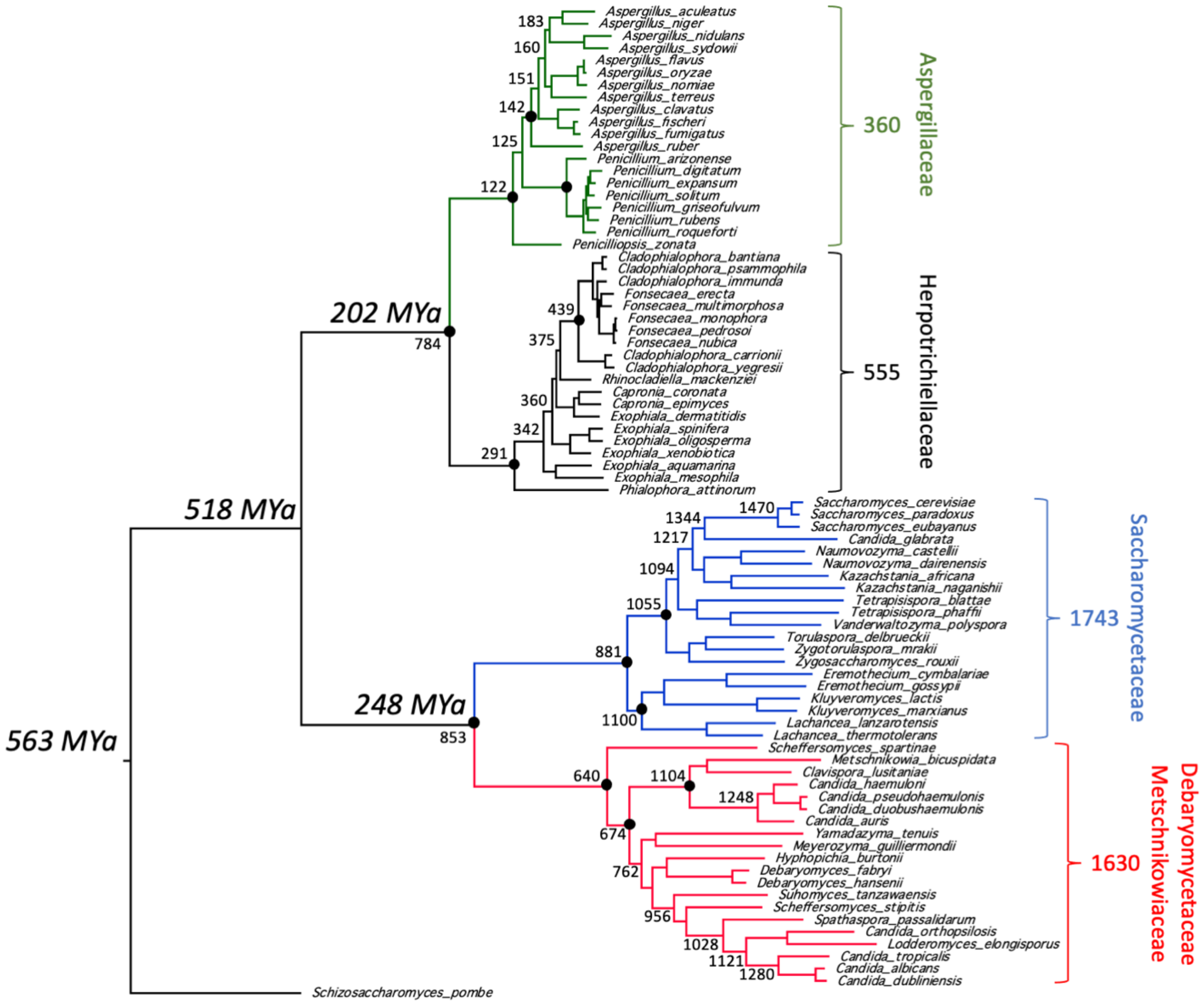

Supplementary Figure 2B. CDI retention within clades. The evolutionary times associated with selected nodes were derived from a recent comprehensive investigation of Ascomycota phylogeny (Shen et al. 2020). Selected nodes with 100% bootstrap support are indicated with black circles. The total numbers of CDIs for each clade are indicated by the taxonomic labels. The numbers associated with selected nodes are the numbers of shared CDIs by all descendants from that node. The numbers of inserts that are shared by the CTG and Saccharomycetaceae clades and by the Aspergillaceae and Herpotrichiellaceae clades are also indicated.

NP_012140_Saccharomyces_cerevisiae/1203-1210	KLTVKIKL
XP_018221496_Saccharomyces_eubayanus/1203-1210	KLTVKIKL
XP_033766929_Saccharomyces_paradoxus/1202-1209	KLTVKIKL
XP_003675109_Naumovozyma_castellii/1170-1177	KLKIKLTM
XP_003668661_Naumovozyma_dairenensis/1196-1203	KLKIKLTL
XP_003959368_Kazachstania_africana/1172-1179	KFKITLKL
XP_022464686_Kazachstania_naganishii/1200-1207	RLRVKLTL
XP_446743_Candida_glabrata/1198-1205	KLKIKLKL
XP_004181716_Tetrapisispora_blattae/1231-1238	KFKVKLTL
XP_003686053_Tetrapisispora_phaffii/1243-1250	SLKVKISL
XP_001646124_Vanderwaltozyma_polyspora/1197-1204	KLKIKLKL
XP_003680552_Torulaspora_delbrueckii/1162-1169	KLKIKLTL
XP_037144276_Zygotorulaspora_mrakii/1188-1195	KLKIRLKL
XP_002494432_Zygosaccharomyces_rouxii/1197-1204	KLKIKLNL
XP_003646975_Eremothecium_cymbalariae/1171-1178	KLKVKISL
NP_985231_Eremothecium_gossypii/1150-1157	KLKVKINL
XP_455284_Kluyveromyces_lactis/1215-1222	KIKLKLNM
XP_022677605_Kluyveromyces_marxianus/1198-1205	KIKLKLNM
XP_022628368_Lachancea_lanzarotensis/1151-1158	RLTVKLSL

Supplementary Figure 3A. Taf14-binding motif in Saccharomycetaceae clade inserts. Basic residues are highlighted in red; hydrophobic residues are highlighted in cyan. NP_012140 is the accession code for Sth1.

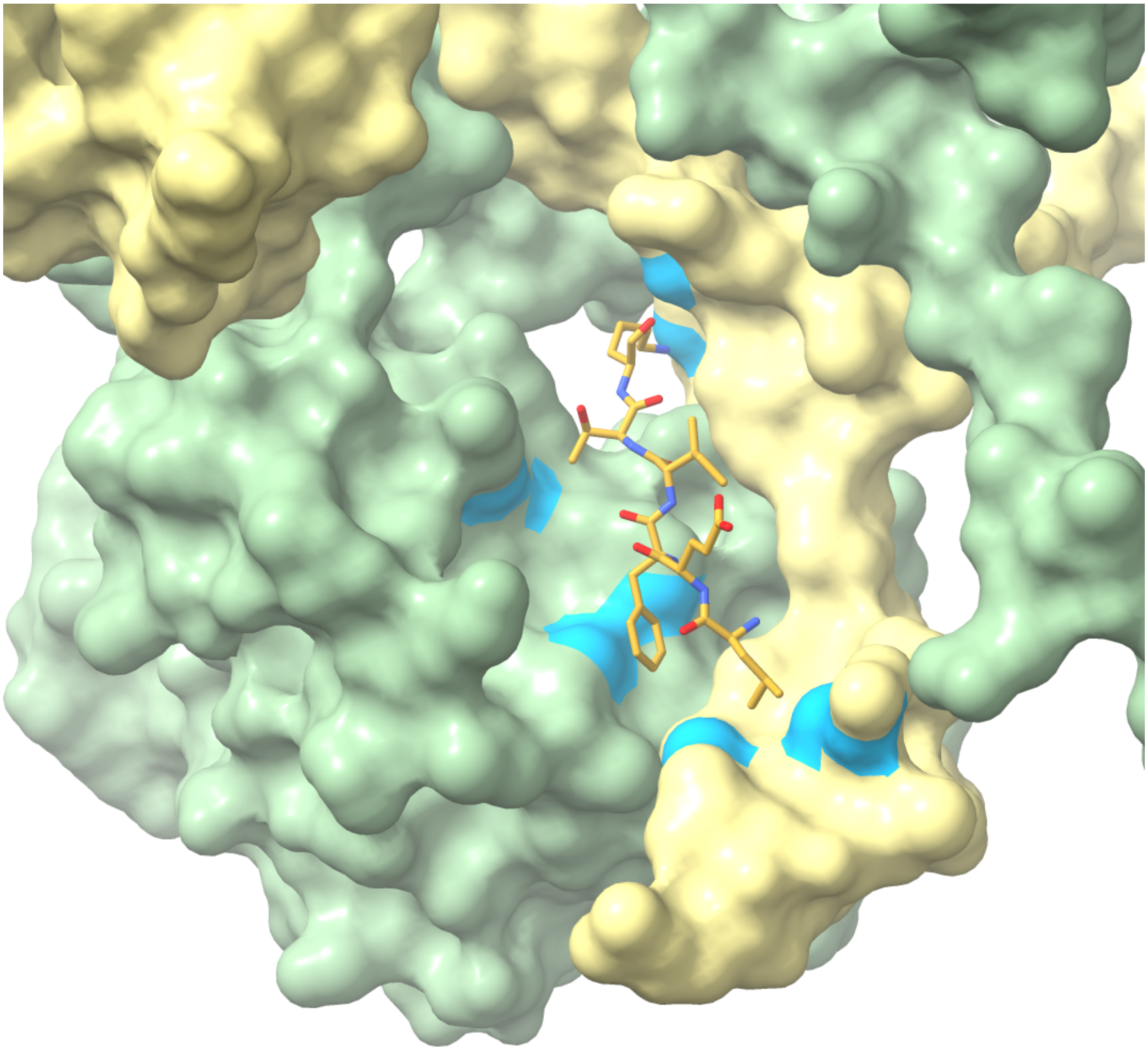

Supplementary Figure 3B. **Sth1 insert 222-227 is located in a pocket formed by RSC2 and RSC58.** The structure accession is PDB:6K15 (Chen et al. 2020)**.** Sth1 residues 222-227 are depicted in gold as a stick model. RSC2 and RSC58 are depicted in surface representation (khaki and green, respectively). Surfaces with 4 Å of residues 222-227 are colored blue.

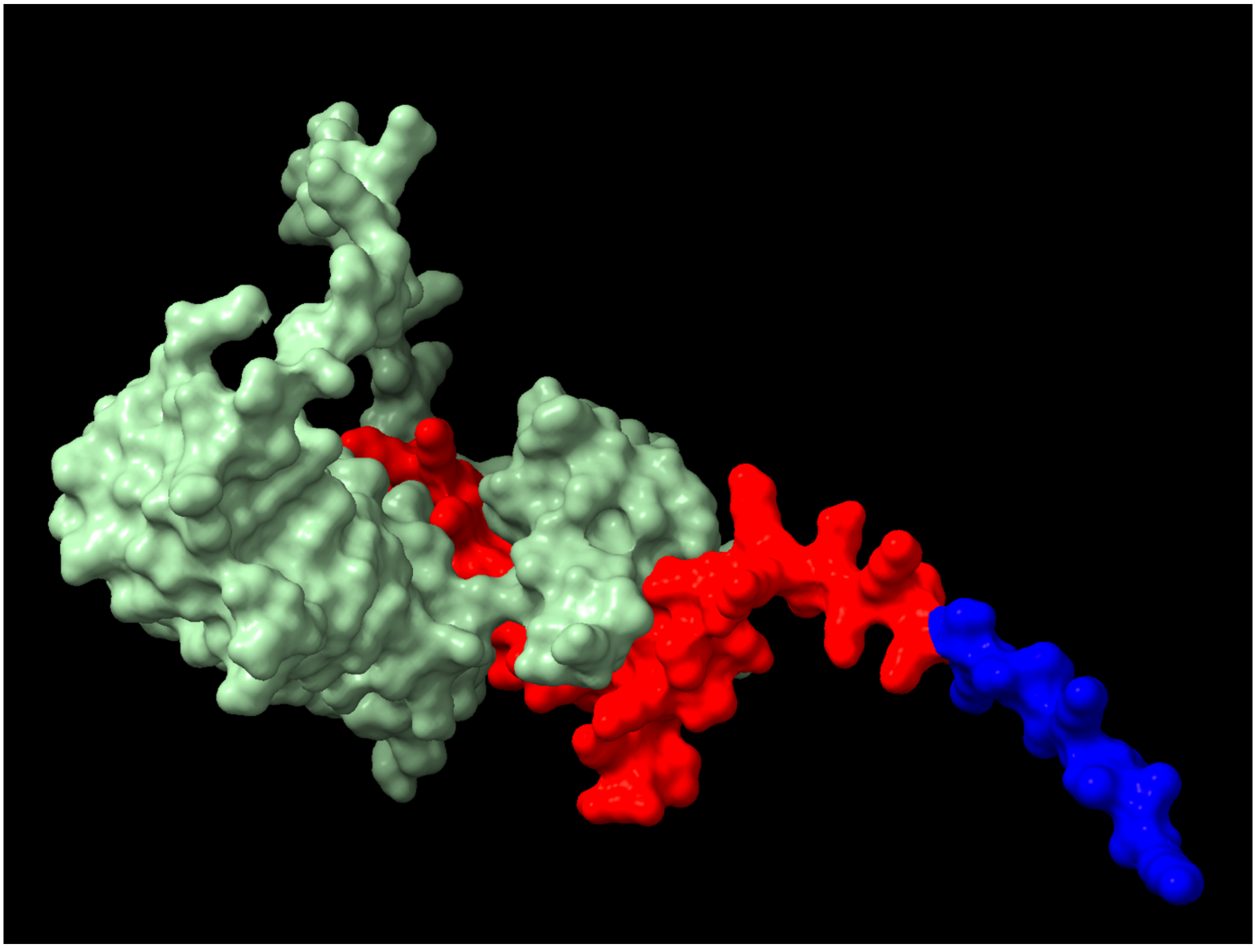

Supplementary Figure 3C. Interaction of the Sth1 Insert Residues 1,183-1,229 with Taf14.

Chains 1.1_A (Taf 14, green) and 1.1_B (Sth1, red and blue) were selected from the ensemble of chains in PDB: 6LQZ for display in a surface representation. The Sth1 insert residues 1,183-1,229 are shown in red.

REFERENCES

Chen G, Wang D, Wu B, Yan F, Xue H, Wang Q, Quan S & Chen Y (2020) Taf14 recognizes a common motif in transcriptional machineries and facilitates their clustering by phase separation. *Nat Commun* 11, 4206.

Shen X-X, Steenwyk JL, LaBella AL, Opulente DA, Zhou X, Kominek J, Li Y, Groenewald M, Hittinger CT & Rokas A (2020) Genome-scale phylogeny and contrasting modes of genome evolution in the fungal phylum Ascomycota. *Sci. Adv.* 6, eabd0079.
